# High Dietary Iron Supplement Induces the Nigrostriatal Dopaminergic Neurons Lesion in Transgenic Mice Expressing Mutant A53T Human Alpha-Synuclein

**DOI:** 10.3389/fnagi.2018.00097

**Published:** 2018-04-06

**Authors:** Fengju Jia, Ning Song, Weiwei Wang, Xixun Du, Yajing Chi, Hong Jiang

**Affiliations:** Department of Physiology, Shandong Provincial Key Laboratory of Pathogenesis and Prevention of Neurological Disorders and State Key Disciplines: Physiology, Medical College of Qingdao University, Qingdao, China

**Keywords:** iron, alpha-synuclein, substantia nigra, Parkinson’s disease, aging

## Abstract

Both alpha-synuclein aggregation and iron deposits are neuropathological hallmarks of Parkinson’s disease (PD). We are particularly interested in whether iron could synergize with alpha-synuclein pathology *in vivo*, especially in the nigrostriatal system. In the present study, we reported transgenic mice with overexpressing human A53T alpha-synuclein, as well as WT mice with high dietary iron displayed hyperactive motor coordination and impaired colonic motility, compared with those with basal dietary iron. Only A53T mice, but not WT mice with high dietary iron exhibited nigral dopaminergic neuronal loss, lower levels of tyrosine hydroxylase (TH) in the substantia nigra (SN) and decreased dopamine contents in the striatum. Although there was no obvious elevation of iron contents in the SN in WT mice with high dietary iron, we observed iron contents in the SN were especially higher than the other brain regions in 12-month aged mice with either high or basal dietary iron. These results suggested high dietary iron supplement could induce nigral dopaminergic neurons lesion in A53T mice, which might be due to the vulnerability of SN to accumulate iron.

## Introduction

Parkinson’s disease (PD) is the second most prevalent neurodegenerative disease, characterized by the selective loss of dopaminergic neurons in the substantia nigra (SN). Both genetic and environmental factors contribute to the degeneration of dopaminergic neurons in PD (Marques and Outeiro, 2013; Campdelacreu, 2014; Wang et al., 2016; Fleming, 2017), although the etiology of PD is still an enigma. The pathological hallmarks of PD are the presence of intracellular inclusions, called Lewy bodies (LB) in the survival dopaminergic neurons (Dauer and Przedborski, 2003; Olanow et al., 2009; Tolleson and Fang, 2013; Dickson, 2018), which are mainly composed by a mixture of substances including alpha-synuclein, ubiquitin, neurofilament proteins and iron (Castellani et al., 2000; Goedert, 2001; Mandel et al., 2004; Fares et al., 2016). Alpha-synuclein, the major driver of dopaminergic neuronal loss, is a central player in the pathogenesis of PD (Olanow and Brundin, 2013; Rocha et al., 2018). As a co-existing factor with alpha-synuclein in LB, iron has been associated with an increased risk of PD (Medeiros et al., 2016; Jiang et al., 2017; Moreau et al., 2018). More noticeably, both neuropathological and imaging studies have demonstrated iron selectively accumulates in the SN of patients or animal model with PD (Martin et al., 2008; Salazar et al., 2008). Three case-control studies with retrospective collection of exposure data also indicated supplemental iron intake was associated with an increase in PD risk (Logroscino et al., 1998; Gorell et al., 1999; Powers et al., 2003). *In vivo* animal models reported high iron dietary supplement induced iron concentrations increase in several brain regions, e.g., cortex, hypothalamus, striatum and SN (Chang et al., 2005; Ke et al., 2005; Qian et al., 2007).

Excessive iron is crucially involved in the formation of reactive oxygen species (ROS), a blanket term for a collection of partially reduced oxygen containing molecules, including superoxide (O_2_•−), peroxides (H_2_O_2_ and ROOH) and free radicals (HO• and RO•) (Dixon and Stockwell, 2014). Iron convey toxicities in the dopaminergic neurons by reacting with H_2_O_2_ produced in the enzymatic process in dopamine metabolism and accelerating the non-enzymatic catalytic process and producing neurotoxic intermediate or end products O_2_•− and ·OH. More recently, ferroptosis was introduced and highlighted to trigger the nonapoptotic cell death dependent on iron and ROS (Stockwell et al., 2017). The potential link between iron and alpha-synuclein was discussed *in vitro* in several studies. Ferrous iron could induce a large increase in α-helix contents of alpha-synuclein by non-specifically binding with its C-terminus domain (Brown, 2007; Bai et al., 2015), and its conformational changes facilitated it prone to aggregate. *In vivo*, the toxicity of mutant alpha-synuclein (A53T and A30P) could be preferentially exacerbated by iron and alpha-synuclein mutation in concert with iron overload could exaggerate selective loss of PPM3 dopaminergic neurons and motor decline in *Drosophila* (Zhu et al., 2016). It is proposed that interaction between iron and alpha-synuclein can cause a vicious circle in PD (Lingor et al., 2017), however, the synergic effect of dietary iron supplement (environmental risk) and alpha-synuclein mutation (genetic factor) on the lesion of dopaminergic neurons in mammals is largely unknown.

In the present study, we adopted the transgenic mouse overexpressing human mutant A53T alpha-synuclein to assess whether high dietary iron supplement might affect nigrostriatal dopaminergic neurons. A53T mice successfully recapitulate prominent motor decline seen in PD patients (Wu et al., 2016), as well as many important non-motor features, e.g., consistent and clinically relevant sleep phenotypes and deterioration of olfactory (Papadimitriou et al., 2016). We also evaluated the iron contents in WT mice with dietary iron supplements to elucidate whether iron accumulated in specific brain regions.

## Materials and Methods

### Ethics Statement

This study was carried out in strict accordance with the recommendations in the Guide for the Care and Use of Laboratory Animals of the National Institutes of Health. The protocol was approved by the Committee on the Ethics of Animal Experiments of Qingdao University. All efforts were made to minimize animal suffering.

### Animal Treatment

Heterozygote A53T transgenic mice (B6; C3-Tg (Prnp-SNCA*A53T)83Vle/J) were originally obtained in breeding pairs from the Jackson Laboratory (004479) to generate a stable breeding colony. Offspring were intercrossed to generate homozygote (A53T) mice and WT mice. C57BL/6 mice were purchased from Vital River Laboratory Animal Technology Co., Limited (Beijing, China). All mice were housed at room temperature under a 12-h light/dark cycle. Food and water were delivered *ad libitum*. Two-month old WT and A53T alpha-synuclein transgenic mice were supplied with high dietary iron for 3 months (the Basal Purified Diet supplied with 2.5% carbonyl iron, PMI catalog# 43784 Purina Mills-Lab Diet^®^ Facility, Richmond, IN, USA) and basal dietary iron (the Basal Purified Diet, PMI catalog# 7024, Purina Mills-Lab Diet^®^ Facility, Richmond, IN, USA) for 3 months (*n* = 6 for each group; Ke et al., 2003). At the end of the dietary iron supplements, rotarod test and pole test were used to detect motor coordination of the mice. And 1-h stool characteristics and frequency assay were applied to assess colonic motility ability. Plasma samples were collected from the angulus oculi medialis of eye in tubes containing ethylene diamine tetraacetic acid (EDTA) as anticoagulant. Then the mice were sacrificed for tissue isolation or perfusion. Twelve-month old C57BL/6 mice were supplied with high dietary iron for 3 months to investigate the effects of aging (*n* = 6 for each group).

### Chemicals and Drugs

The primary antibodies against tyrosine hydroxylase (TH) were purchased from Merck Millipore (Darmstadt, Hesse, Germany). The Alexa Fluor^®^ 488 donkey anti-mouse IgG were from Invitrogen (Carlsbad, CA, USA). Anti-rabbit secondary antibodies were from Santa Cruz Biotechnology (Dallas, TX, USA). All other chemicals and regents were of the highest grade available from local commercial sources.

### Rotarod Test

Motor coordination and balance were assessed using a rotarod apparatus (Med Associates, Fairfax, VT, USA). The animals were placed on the rolling rod with an initial speed of 4 rpm and an accelerating speed levels (4–40 rpm) mode of the apparatus. Then, two trials with an interval trial time of 1 h were performed. The mean latency to fall off the rotarod was recorded.

### Pole Test

The pole test has been used to detect bradykinesia and motor coordination in PD mice. The mice were placed facing upwards at the top of a wooden pole (50 cm long and 1 cm in diameter) that led into their home cage. The mice were trained to turn to orient downward and traverse the pole into their home cage. Then, the amount of time to turn to orient downward was tested. Five trials were performed with each mouse across the trials. Time was limited to 60 s.

### 1-h Stool Characteristics and Frequency Assay

1-h stool characteristics and frequency assay were performed between 10:00 and 12:00 on each day. Each animal was removed from its home cage and placed in a clean, clear plastic cage without food or water for 1 h. Stools were collected immediately after expulsion (to avoid evaporation) and placed in sealed tubes. Tubes were weighed to obtain the wet weight of the stool, which was then dried overnight at 65°C and reweighed to obtain the dry weight. The water content of stool (%) was calculated by (wet weight − dry weight)/wet weight * 100. Wet weight of stool, dry weight of stool, the water content of stool and stool frequency were observed throughout the collection period.

### TH Immunofluorescence Staining

Brains were removed and postfixed in 4% paraformaldehyde for 6 h, then, transferred to 30% sucrose until sectioning. Sections (20 μm) were cut on a freezing microtome (Leica, Wetzlar, Hesse-Darmstadt, Germany). Alternate SN sections were stained for TH. After being washed three times in phosphate buffer saline (PBS) plus 0.3% Triton X-100, sections were incubated overnight with primary antibody of TH (1:2000) at 4°C. Then washed three times with PBS and incubated in the second antibody of Alexa Fluor^®^488 donkey anti-mouse IgG at room temperature. Next, sections were rinsed with PBS for three times. Sections were mounted with 70% glycerin. The numbers of TH^+^ in the SN were estimated using MBF Stereo Investigator software (MBF Bioscience). The stereologist was blind to the treatment received. One of every six serial sections of the mid-brain were counted to cover the entire mouse. Stereological details were as follows: counting grid, 240 μm × 180 μm; counting frames, 80 μm × 60 μm.

### Western Blotting Analysis

The brain tissues from SN were lysed using lysis buffer (50 mmol/L Tris-HCl, 150 mmol/L NaCl, 1% Nonidet-40, 0.5% sodium deoxycholate, 1 mmol/L EDTA and 1 mmol/L phenylmethanesulfonyl fluoride) with a protease inhibitor cocktail, and the protein concentration was determined by the Bradford assay kit (Bio-Rad Laboratories, Hercules, CA, USA). Twenty micrograms of total protein from each sample was separated using 8% sodium dodecyl sulfate–polyacrylamide gels and then transferred by electroblotting to polyvinylidene difluoride membranes. After 2 h of blocking with 10% nonfat milk at room temperature, the membranes were incubated with primary antibody against TH (1:2000) overnight at 4°C. Anti-rabbit secondary antibodies conjugated to horseradish peroxidase was used at 1:10,000. Blots were also probed with anti-β-actin monoclonal antibody (1:10,000) as a loading control. Cross-reactivity was visualized using enhanced chemiluminescence western blotting detection reagents and analyzed by scanning densitometry with a Tanon Image System (Tanon, Shanghai, China).

### High-Performance Liquid Chromatography With Electrochemical Detection (HPLC-ECD)

Both sides of the striatum were isolated carefully and transferred to liquid nitrogen. Samples were weighed and then homogenized in 0.3 ml liquid A (0.4 M perchloric acid). After initial centrifugation (120,000 rpm for 20 min at 4°C), 80 μl of the supernatant was transferred into Eppendorf tubes, and 40 μl liquid B (20 mM citromalic acid potassium, 300 mM dipotassium phosphate, 2 mM EDTA·2Na were added. After additional centrifugation (120,000 rpm for 20 min at 4°C), 100 μl of the supernatant was assayed for DA by HPLC. Separation was achieved on a PEC18 reversed-phase column. The mobile phase (20 mM citromalic acid, 50 mM sodium caproate, 0.134 mM EDTA·2Na, 3.75 mM sodium octane sulfonic acid, 1 mM di-sec-butylamine and containing 5% (v/v) methanol) was used at a flow-rate of 1 mL/min. A 2465 electrochemical detector (Waters, Milford, MA, USA) was employed and was operated in screen mode.

### Iron Content Measurements

Inductively coupled plasma mass spectrometer (ICP-MS) 7500CE (Agilent, Santa Clara, CA, USA) was used for the determination of the iron content of different tissues. Before the measurement, tissue samples were digested in order to destruct tissues and proteins and obtain a limpid solution. According to the sample, between 10 mg and 105 mg of tissue was available for digestion. Two milliliter aliquot of nitric acid (65%) and 1.5 ml of hydrogen peroxide (35%) were added. The mixture was placed in a Milestone MS-200 microwave oven and exposed, for brain samples, at 250 W for 2 min, 0 W for 2 min, then 250 W for 6 min and 650 W for 5 min and 5 min of ventilation. For liver and spleen samples, the digestion program was slightly different: 3 min at 250 W, 30 s at 0 W, then 5 min at 250 W, 30 s at 0 W, 5 min at 450 W, 30 s at 0 W, 5 min at 600 W and 25 min of ventilation. After 20 min of cooling at room temperature, samples were ready for iron content determinations.

### Statistical Analysis

All analyses were performed in SPSS 19.0 (IBM, Chicago, IL, USA). Data were expressed as mean values ± SEM. Independent sample *t*-test was adopted to analyze the difference between the high dietary iron and basal dietary iron groups. One-way analysis of variance (ANOVA) analysis was applied to assess the iron contents between the different brain regions. *P* < 0.05 was considered to be statistically significant.

## Results

### Hyperactivity of Motor Coordination in Both A53T Mice and WT Mice With High Dietary Iron

**Figure 1:**
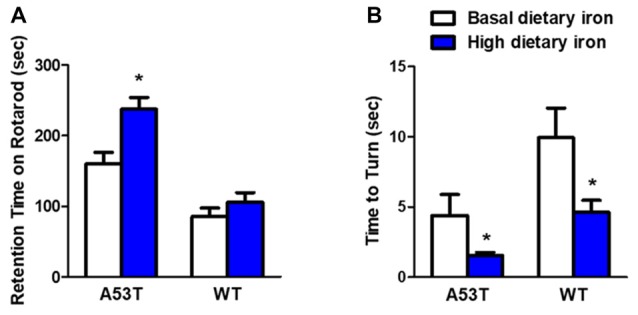
Changes of motor coordination ability in A53T and WT mice with high dietary iron. In A53T mice, the residence time was longer **(A)**, and the time to turn to orient down-ward was shorter **(B)** in high dietary iron group compared to the basal dietary iron group. In WT mice, although there was a tendency of the latency to escape to be delayed in rotarod test in high dietary iron group, no significant difference was observed when compared to the basal dietary iron group **(A)**. The high dietary iron group showed a significant decrease in the time to turn to orient down-ward **(B)** in the pole test, compared with the basal dietary iron group. Each bar represented the mean ± SEM. *P < 0.05, compared with the corresponding basal dietary iron group, *n* = 6.

We first evaluated the motor coordination ability in A53T mice with high or basal dietary iron. The residence time on rotarod treadmills was delayed by 48.3% in A53T mice with high dietary iron, compared with that of A53T mice with basal dietary iron (Figure 1A). In the pole test, the time to turn to orient down-ward in A53T mice with high dietary iron was 64.5% shorter than that of A53T mice with basal dietary iron (Figure 1B). These results indicated that there was hyperactivity of motor coordination ability in A53T mice with high dietary iron when compared with those with basal dietary iron.

Compared with WT mice with basal dietary iron, the residence time was unchanged in the rotarod test in the WT mice with high dietary iron (Figure 1A), however, they showed a significant 53.3% decrease in the time to turn to orient down-ward in the pole test (Figure 1B). These results indicated that motor coordination ability was also abnormal in WT mice with high dietary iron when compared with WT mice with basal dietary iron.

### Impaired Colonic Motility in Both A53T Mice and WT Mice With High Dietary Iron

**Figure 2:**
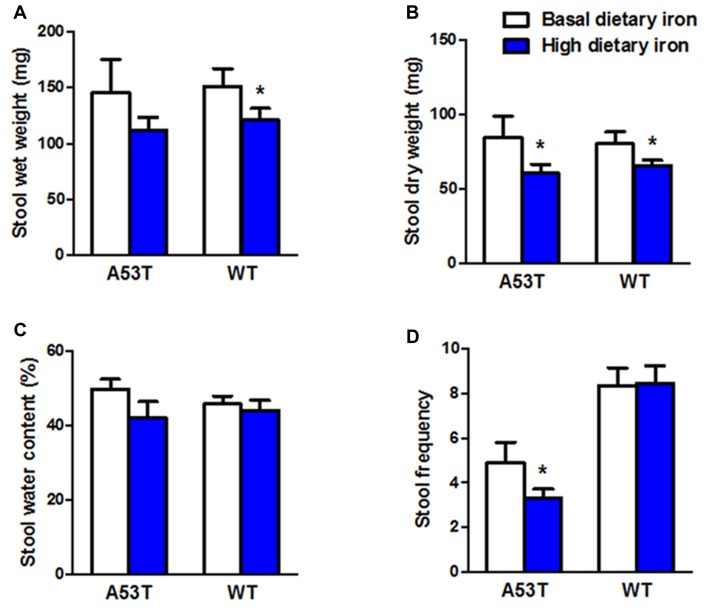
Changes of colonic motility in A53T and WT mice with high dietary iron. The colonic motility was observed in A53T mice and WT mice. In A53T mice, dry weight of stool **(B)** and stool frequency **(D)** in the high dietary iron group were lower than the basal dietary iron group, although no changes were observed in wet weight of stool **(A)** and water content of stool **(C)**. In WT mice, compared to the basal dietary iron group, the high dietary iron group showed decreased wet weight of stool **(A)** and dry weight of stool **(B)**, although there were no variations either in water content of stool **(C)** or stool frequency **(D)**. Each bar represented the mean ± SEM. *P < 0.05, compared with the corresponding basal dietary iron group, *n* = 6.

In addition to the motor coordination, we evaluated the colonic motility in A53T mice with high or basal dietary iron. Dry weight of stool, and stool frequency in A53T mice with high dietary iron were lower than those of A53T mice with basal dietary iron (Figures 2B,D) by 28.7% and 26.7%, respectively, although wet weight of stool and stool water content were unchanged (Figures 2A,C). These results indicated the colonic motility was abnormal in A53T mice with high dietary iron.

Wet weight and dry weight of stool in WT mice with high dietary iron was 20.0% and 18.2% lower than that of WT mice with basal dietary iron, respectively (Figures 2A,B). Unlike the A53T mice, WT mice did not show any changes in stool frequency (Figure 2D).

### Nigrostriatal Lesion in A53T Mice but Not WT Mice With High Dietary Iron

**Figure 3:**
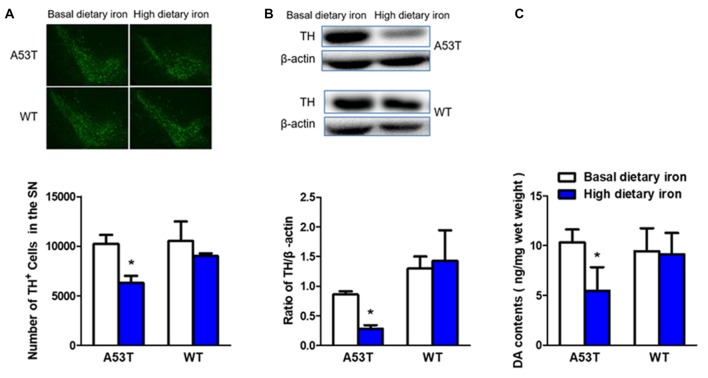
Changes of dopaminergic neurons of A53T and WT mice with high dietary iron. In A53T mice, the number of dopaminergic neurons in substantia nigra (SN) **(A)**, level of tyrosine hydroxylase (TH) protein in SN** (B)** and DA contents in striatum **(C)** were lower in high dietary iron group compared to the basal dietary iron group. However, no changes were detected in the number of dopaminergic neurons in SN **(A)**, level of TH protein in SN** (B)** and DA contents in striatum **(C)** in WT mice. Each bar represented the mean ± SEM. *P < 0.05, compared with the corresponding basal dietary iron group, *n* = 6.

The number of dopaminergic neurons in the SN was 38.5% lower in A53T mice with high dietary iron than that of A53T mice with basal dietary iron (Figure 3A). Similar results were found in the TH protein levels in the SN (67.3% decrease, Figure 3B). Next, we measured the DA contents in the striatum to better determine the function of the nigral dopaminergic neurons. DA content of A53T mice with high dietary iron was 47.0% lower than that of A53T mice with basal dietary iron (Figure 3C).

However, no changes were observed in whatever dopaminergic neurons number (Figure 3A), TH protein levels in the SN (Figure 3B) or DA contents in the striatum in WT mice with high dietary iron (Figure 3C). These results implied that high dietary iron could induce nigrostriatal dopaminergic neurons lesion in A53T mice but not WT mice.

### SN Was Extremely Susceptible for Iron Accumulation During Aging

**Figure 4:**
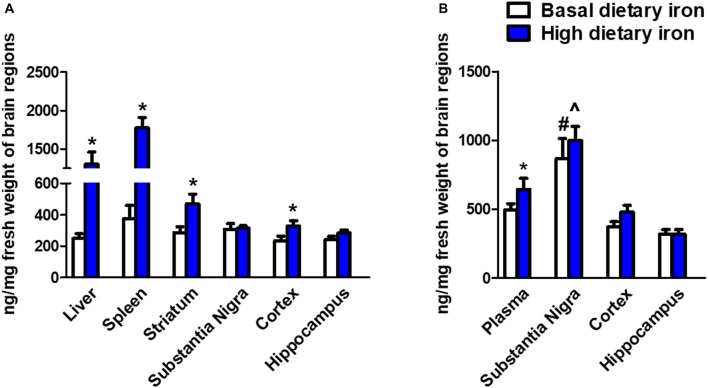
Iron contents in peripheral organs and brain regions in 2-month and 12-month mice with dietary iron supplement for 3 months. **(A)** Iron contents in peripheral organs and brain regions in 2-month mice with dietary iron supplement: Iron contents were increased in the liver and spleen (peripheral organs) in 2-month WT mice with high dietary iron supplement for 3 months. Although no changes were observed in the other brain regions, iron contents were elevated in the cortex and striatum of 2-month mice with high dietary iron for 3 months. Each bar represented the mean ± SEM. *P < 0.05, compared with the corresponding basal dietary iron group; *n* = 6. **(B)** Iron contents in peripheral organs and brain regions in 12-month mice with dietary iron supplement: Iron contents were increased in the plasma. Iron contents in the SN were obviously higher than the other brain regions in 12-month mice with basal dietary iron and high dietary iron for 3 months. Each bar represented the mean ± SEM. *P < 0.05, compared with the corresponding basal dietary iron group; ^#^*P* < 0.05, compared with the other brain regions of mice with basal dietary iron; ^∧^*P* < 0.05, compared with the other brain regions of mice with high dietary iron; *n* = 6.

We next investigated whether the dietary iron supplements could trigger iron deposits in specific brain regions. As we expected, iron deposit was observed in the peripheral tissues like liver and spleen (Figure 4A). This indicated the dietary iron supplements for 3 months might cause systemic iron overload. The nigrostriatal system was then analyzed and we observed a dramatic 58.7% increase in the striatum of 2-month mice with high dietary iron for 3 months when compared with that of mice with basal dietary iron (Figure 4B). However, there were no changes observed in the SN. Unexpectedly, there was a 39% increase of iron content in the cortex. We then analyzed whether iron contents showed a similar pattern in the 12-month mice with high dietary iron for 3 months. Elevated plasma iron levels were observed. Overall there were no obvious changes in the iron contents in the SN, cortex and hippocampus in the high dietary iron group when compared with those of mice with basal dietary iron. However, it was quite interesting that iron contents of the SN in the 12-month mice were extremely higher than those of the other brain regions investigated (Figure 4B). This phenomenon was applied to both the high dietary iron supplemented group and basal dietary iron group. It was noteworthy that SN was the most susceptible brain region for iron accumulation during aging (iron contents were doubled in the 12-month mice compared to those in the 2-month mice regardless of dietary iron).

## Discussion

To our knowledge, this is the first report of the lesion of nigral dopaminergic neurons in A53T mice with high dietary iron supplement *in vivo*. The synergic effect of dietary iron supplement and A53T alpha-synuclein resulted in a loss of dopaminergic neurons, decreased TH protein levels in the SN and DA contents in the striatum. We also demonstrated SN was subject to accumulate iron with aging.

The interaction of iron and alpha-synuclein *in vitro* was investigated in several studies (Belaidi and Bush, 2016; Lingor et al., 2017). Alpha-synuclein binds to iron possibly via electrostatic interactions (Liu and Franz, 2005). Furthermore, S129 phosphorylation was shown to increase affinity of iron binding in peptide studies (Liu and Franz, 2007). The binding of iron could in turn induce a large increase in α-helix contents of alpha-synuclein, making it more prone to aggregate (nearly 50 fold; Uversky et al., 2001). The structural link between iron and alpha-synuclein was proposed by the presence of iron responsive element in the alpha-synuclein 5’-untranslated region, indicating alpha-synuclein expression was modulated at the posttranscriptional level by iron (Friedlich et al., 2007). As a ferrireductase, alpha-synuclein may intensify Fe^2+^ accumulation by converting Fe^3+^ to Fe^2+^ (Brown, 2013). The excess generation of Fe^2+^ could interact with other proteins or lipids and generate radicals through the Fenton reaction and eventually lead to cell damage. We supposed high alpha-synuclein levels in A53T mice might facilitate iron retention given iron and alpha-synuclein co-localized and interacted in the A53T mice supplied with high iron diet.

We are particularly interested in the dopaminergic neurons also because alpha-synuclein is believed to be involved in multiple steps in dopamine metabolism. First, one of the vital functions of dopaminergic neuron in the SN is the synthesis of dopamine via the enzyme TH. It was reported the increased alpha-synuclein not only inhibited TH activity (Kaushik et al., 2007; Liu and Franz, 2007), but also modulated dopamine packaging into vesicles and subsequent vesicle release from neurons (Venda et al., 2010; Oaks and Sidhu, 2011). In addition, dopamine could form a heterodimer with alpha-synuclein (McDowall and Brown, 2016). What’s more, dopamine inhibited the fibrillation of alpha-synuclein into amyloid, and promoted alpha-synuclein aggregation into SDS-stable soluble oligomers (Pham and Cappai, 2013). This dopamine and alpha-synuclein interactions promoted the vulnerability of dopaminergic neuron in A53T mice with basal dietary iron, although no dopaminergic neurons degeneration was observed in these mice. However, A53T mice, but not WT mice with high dietary iron exhibited damaged nigrostriatal function, further suggesting synergistic effects of iron and alpha-synuclein.

Consistent with our recent publication (Wang et al., 2018), we observed distinct hyperactive motor coordination ability in both A53T and WT mice with dietary iron supplement, although there was no nigrostriatal damage in the WT mice. In fact, this kind of hyperactivity we observed was in accordance with previously reported increased locomotor activity before the onset of motor dysfunction in A53T transgenic mice (Unger et al., 2006; Wu and Hallett, 2013). We thought this kind of hyperactivity was a compensatory effect of cerebellum and the compensatory effect helped the mice maintain better motor performance (Wu et al., 2011). We also observed the impaired colon activities in both A53T and WT mice with dietary iron supplement. Gastrointestinal (GI) dysfunction is the one of the most common non-motor symptoms of PD and reported in 80%–90% patients afflicted with this common neurodegenerative disorder (Barichella et al., 2009; Barone et al., 2009; Noorian et al., 2012; Goldman and Postuma, 2014). A53T alpha-synuclein mice developed age-related declines in stool frequency and gastric emptying consistent with those seen in human PD (Noorian et al., 2012). One clinical study reported that synucleinopathy in the enteric nervous system was significantly correlated with frequency and severity of GI dysfunction motor disability in PD patients (Mrabet et al., 2016), supporting the relationship between alpha-synuclein deposition in the enteric nervous system and GI dysfunction. In the present study, the GI dysfunctions are taken in account considering that the dietary iron supplements might induce and interact synucleinopathy on this primary site.

However, we did not observe the iron retention in the SN in mice with high dietary iron supplement, although the findings in the peripheral organs showed that high dietary iron for 3 months caused an iron-overload state. This was different from the observations made by Qian et al. (2007). They found the total iron contents in the SN were higher in SD rats (21 days of age) fed with the basal purified diet supplied with 2.5% carbonyl iron for 8 weeks. We supposed that the animal age might contribute to the difference. We did observe iron contents were first elevated in the striatum of 2-month mice with high dietary iron. It was reported that the blood brain barrier (BBB) of the striatum was especially fragile to ischemic, osmotic, or other stressors. Besides, PD patients showed a loss of integrity of the BBB in the striatum (Gray and Woulfe, 2015). Based on these evidences, we hypothesized that the changed BBB permeability during iron overloaded condition might be responsible for the iron accumulation in this area. This was in accordance with the fact that iron aggregation was initiated at the levels of axon terminals (Gerlach et al., 1994). Therefore, it was reasonable that iron was accumulated first in the striatum and then retrograde transported to the SN. Unexpectedly, iron deposit in mice with iron supplements was observed quite early in the cortex. We previously reported that different iron regulatory mechanisms might exist and iron redistribution may occur between the cortex and the SN of patients with PD (Yu et al., 2013), therefore it is worthy of revealing the cortex iron metabolism in the future.

It was interesting that in the elderly mice (12-month), the SN was the prominent area that had ample iron content, although there were no differences in iron contents after dietary iron supplement. This indicated that SN was susceptible to iron accumulation in the elderly mice. Kaur et al. (2007) found that increased neonatal iron feeding induced significant abnormality of behavior and depletion of striatal DA in the aging rats rather than those young ones (Chen et al., 2015). This is in accordance with a similar report that infantile exposure to environmental metal lead (Pb) was linked to Alzheimer’s disease-like pathology in aged monkeys (Wu et al., 2008). We reported that high dietary iron supplement induced iron deposits early in the striatum in the young mice, meanwhile, aging induced iron deposits preferentially in the SN in the elderly mice. We speculated iron exposure might exert long-term outcomes and aging was a critical risk factor for iron accumulation in the nigrostriatal system.

In conclusion, our data supported that high dietary iron supplement could induce severe disease phenotypes, particularly the lesion of nigral dopaminergic neurons, in A53T mutant alpha-synuclein mice. SN was susceptible to iron accumulation during aging, supporting the multiple hits by iron, mutant alpha-synuclein and aging contribute to the vulnerability of dopaminergic neurons in PD.

## Author Contributions

HJ conceived and designed research. FJ and YC performed experiments. NS and XD analyzed data. FJ, NS and WW wrote the manuscript.

## Conflict of Interest Statement

The authors declare that the research was conducted in the absence of any commercial or financial relationships that could be construed as a potential conflict of interest.
